# Gender and urban-rural influences on antibiotic purchasing and prescription use in retail drug shops: a one health study

**DOI:** 10.1186/s12889-023-15155-3

**Published:** 2023-02-02

**Authors:** Emily K. Rousham, Papreen Nahar, Mohammad Rofi Uddin, Mohammad Aminul Islam, Fosiul Alam Nizame, Nirnita Khisa, S. M. Salim Akter, Mohammad Saeed Munim, Mahbubur Rahman, Leanne Unicomb

**Affiliations:** 1 School of Sport, Exercise and Health Sciences, Loughborough University, LE11 3TU Loughborough, Leicestershire, UK; 2 Department of Global Health and Infection, Brighton and Sussex Medical School, University of Sussex, Brighton and Hove, UK; 3 Environmental Interventions Unit, Infectious Diseases Division, International Centre for Diarrhoeal Disease Research, Bangladesh (icddr,b), Dhaka, Bangladesh; 4 Paul G. Allen School for Global Health, Washington State University, Pullman, WA USA; 5 Tarum Development Organization, Rangamati, Bangladesh

**Keywords:** Drug resistance, Antibiotics, Community health services, Low income and middle-income countries, Pharmacy, Observation, Gender equality and social inclusion (GESI), Prescription, Drug shop

## Abstract

**Introduction:**

Few studies have reported antibiotic purchases from retail drug shops in relation to gender in low and middle-income countries (LMICs). Using a One Health approach, we aimed to examine gender dimensions of antibiotic purchases for humans and animals and use of prescriptions in retail drug shops in Bangladesh.

**Methods:**

We conducted customer observations in 20 drug shops in one rural and one urban area. Customer gender, antibiotic purchases, and prescription use were recorded during a four-hour observation (2 sessions of 2 hours) in each shop. We included drug shops selling human medicine (n = 15); animal medicine (n = 3), and shops selling both human and animal medicine (n = 2).

**Results:**

Of 582 observations, 31.6% of drug shop customers were women. Women comprised almost half of customers (47.1%) in urban drug shops but only 17.2% of customers in rural drug shops (p < 0.001). Antibiotic purchases were more common in urban than rural shops (21.6% versus 12.2% of all transactions, p = 0.003). Only a quarter (26.0%) of customers who purchased antibiotics used a prescription. Prescription use for antibiotics was more likely among women than men (odds ratio (OR) = 4.04, 95% CI 1.55, 10.55) and more likely among urban compared to rural customers (OR = 4.31 95% CI 1.34, 13.84). After adjusting for urban-rural locality, women remained more likely to use a prescription than men (adjusted OR = 3.38, 95% CI 1.26, 9.09) but this was in part due to antibiotics bought by men for animals without prescription. Customers in drug shops selling animal medicine had the lowest use of prescriptions for antibiotics (4.8% of antibiotic purchases).

**Conclusion:**

This study found that nearly three-quarters of all antibiotics sold were without prescription, including antibiotics on the list of critically important antimicrobials for human medicine. Men attending drug shops were more likely to purchase antibiotics without a prescription compared to women, while women customers were underrepresented in rural drug shops. Antibiotic stewardship initiatives in the community need to consider gender and urban-rural dimensions of drug shop uptake and prescription use for antibiotics in both human and animal medicine. Such initiatives could strengthen National Action Plans.

**Supplementary Information:**

The online version contains supplementary material available at 10.1186/s12889-023-15155-3.

## Background

Drug shops are recognised as an important source of access to medicines including antibiotics [1, 2]. In low- and middle-income countries (LMICs), drug shops offer affordable and accessible healthcare and fill an important gap in under-resourced public healthcare systems [3, 4]. Drug shop staff have been described as ‘de facto’ primary health care providers [4]. Whilst there is global emphasis on reducing the inappropriate use of antibiotics, retail pharmacies and informal drug shops continue to play a major role in healthcare provision and form an essential component of Universal Health Coverage [3].

Over-the-counter provision of antibiotics leads to inappropriate use of antibiotics which, in turn, is considered a key driver of the emergence of antibiotic resistance worldwide [5]. Over-the counter sales lead to overuse of antibiotics for mild or non-bacterial infections and are more likely to be of inappropriate dose, duration and more likely to use higher generation antibiotics for humans [6]. Studies across south and south-east Asia reveal a high proportion of antibiotics sold without prescription. In Vietnam, 88% (urban) and 91% (rural) of sales in a survey of 17 pharmacies were without prescription [7]. A study in Bangalore, India, found 66.7% of pharmacies (174 out of 261 observed) sold antimicrobial drugs without a prescription [8], although another urban survey in urban Bengaluru, India, reported lower rates of 33% of chain stores and 42% of independent pharmacies selling antibiotics without a prescription [9]. In Sri Lanka, 1 in 3 community pharmacies dispensed antibiotics without prescription [10]. A scoping review of antibiotic use in south-east Asia found that research on gender differences is scarce, but suggested that these will intersect with contextual factors such as education and socioeconomic status [11]. Studies of prescribing and dispensing practices for humans have frequently employed mystery shoppers or simulated clients [9, 12–15]. However, such studies do not provide insights into the attributes and purchasing practices of genuine customers.

Bangladesh, an upper-middle income country based on the human development index [16], has a thriving market economy of retail drug shops. Many drug shops operate without a license and staff have minimal training [17]. In a study of antibiotic sales in six countries, Bangladesh and Vietnam had the highest proportion of non-licensed antibiotic dispensing outlets [18]. Over half (52%) of 156 surveyed antibiotic suppliers in a rural site in Matlab, southern Bangladesh, were not licensed [18, 19]. In Bangladesh, government policy prohibits the sale of antibiotics without a prescription [20] and the legal requirement is for staff in drug shops to have a minimum Grade C (certificate) pharmacist qualification. In 2016, the government introduced a pilot initiative to register model pharmacies (requiring a minimum Grade A pharmacist MPharm or BPharm qualification), and model medicine shops (minimum Grade C pharmacy qualification), known as the Bangladesh Pharmacy Model Initiative (BPMI), however this did not progress to national roll out.

The WHO recommends that gender and equity considerations are included in National Action Plans on antibiotic resistance to strengthen actions to prevent AMR [21]. There is limited evidence, however, on differences between men and women on antibiotic use and consumption, and ‘even less in terms of a comprehensive gender analysis of how gender norms, roles and relations affect antibiotic use’ [21]. Health systems research can also be better informed on uptake of drug shop services and patterns of antibiotic purchasing and prescription use through use of a gender lens [22]. However, data from LMICs are mostly lacking. In this study, we consider gender, a socially constructed identity, as encompassing the social norms, roles, behaviours and attributes of men and women [23] in the context of Bangladesh. This study therefore aimed to examine the use of drug shops for antibiotic purchasing and prescription use by men and women in urban and rural settings. This formed part of a wider study that examined healthcare seeking behaviours for antibiotics and dispensing of antibiotics for humans and animals by qualified and unqualified prescribers [24, 25], To incorporate a One Health perspective which is an integrated, unifying approach that aims to sustainably balance and optimize the health of people, animals and ecosystems [26], we included drug shops that sold antibiotics for humans, for animals and shops selling medicines for both humans and animals.

## Methods

### Study site and recruitment

We conducted the study in a rural area of Mirzapur sub-district (*Upazila*) in Tangail district and the urban town centre in Tongi sub-district, Gazipur district. In both locations, we selected areas that had not been part of previous community-based interventions or health surveys. Both areas were served by public health facilities (community clinics) but only the urban area had a government hospital. Private healthcare providers (qualified and unqualified) and diagnostic centres were also common in both the rural and urban area. Drug shops were available throughout the study areas in local shopping areas and markets [25, 27]. Prior to data collection, the study team visited the two areas for familiarisation with the community leaders and stakeholders. The research team visited key personnel and government authorities (local government health complex and local government livestock office in the rural site, and government health department in the city corporation of the urban site) to explain the purpose of the study and seek agreement for the study to go ahead. The observation process was piloted in one rural drug shop (for human medicine) for training purposes and to refine the data collection tool. The first phase of data collection took place from October-November 2017 (8 shops). A second period of fieldwork was conducted in September 2019 (12 shops) to increase the number of customer interactions that were observed across the different types of drug retail shops. The time interval between the two rounds of data collection were due to staffing constraints while processing and analysis of other components of the research project took place [25, 27].

### Training and recruitment

Prior to data collection, team members were trained through a 14 day classroom and field training workshop on qualitative research methods and AMR including customer observations by an experienced medical anthropologist (PN). The field team comprised one female and two male researchers at icddr,b, who had relevant Masters’ training and fieldwork experience in Bangladesh. Data collection, fieldwork, data entry and cleaning were overseen by the research manager (FN) [25].

We recruited drug shops purposively using snowball sampling. Research staff started the selection of drug shops by conducting a transect walk and noting the available drug shops along the roads leading away from the government health facility (sub-district health complex) in both the rural and urban site. Word-of-mouth recommendations from local residents were used to ask which drug shops were either popular or were considered to have experienced/knowledgeable staff, or shops that residents would recommend in their area. Using these suggestions, recruitment took place by the researchers approaching drug shop owners directly. As formative research, we did not conduct a power calculation. We used quota sampling to recruit an equal number of shops in the urban and rural area (50% of the total in each area). We also aimed to purposefully recruit up to five shops in each area selling both human and animal medicine or animal medicine only but were unable to meet this target in the urban site. The drug shop owners were approached prior to any data collection to ask whether they would be willing to take part in the study. If they agreed, a date was arranged for the observations to take place in their shop. Of all the shop owners approached to participate, one rural shop declined due to concerns that there was insufficient space, and that business would be adversely affected.

### Ethical approval

Ethical approval for the study was granted by the Institutional Review Board at icddr,b (PR-16,100) and Loughborough University (R17-P081). Participants were provided with a written participant information sheet in Bengali which was read aloud to them, and they were given an opportunity to ask questions before signing a written informed consent form in Bengali. All participation was voluntary.

### Drug shop observations

Two observation periods per shop took place, each of 2 hours’ duration. One session took place in the morning (approximate timing 9-11am or 10-12am, depending on the shop opening times) and one in the late afternoon or early evening period (approximately 4-6pm) to capture variability at different times of day. Shops and market stalls commonly closed in the middle of the day hence no observations were scheduled at this time.

The researcher sat in the shop where they could observe without intruding in normal business. Observations of transactions were recorded on a data collection sheet which included structured sections for completion (observed gender of customer, use of a doctor’s prescription, names of medicines including antibiotics purchased) and blank spaces to supplement this information with explanatory or supplementary notes. Before leaving, the researcher asked the customer whether they were the patient or whether the medicines were bought for another person/patient (by proxy) or for an animal or livestock. Activities were recorded in real time. If more than one customer was being served simultaneously, the objective was to follow one transaction from start to finish rather than to observe all transactions taking place.

### Data analysis

Data were recorded by hand then entered into a database in Microsoft Excel, cleaned and coded. Original written notes were referred to for clarification regarding the nature of the shops or transactions. Medicines were identified from the brand name on the packaging which were then checked by a clinically trained researcher who converted to generic names based on the details provided on the relevant pharmaceutical company websites. Customers were categorised as purchasing medicine for themselves (patient), a family member or an animal. Some customer purchases were made by a neighbouring drug shop seller, or healthcare provider (e.g., rural medical practitioner (RMP) or doctor) and were coded as such.

We report frequencies of prescription use for antibiotics as number (n) and percentages and compared these for gender (male, female) or urban versus rural location. Descriptive statistics are presented using chi-square tests and the likelihood of prescription use was estimated with adjusted and unadjusted odds ratios (OR) with 95% confidence intervals (CI) from univariable and multivariable logistic regression analyses respectively. Statistical analyses were conducted using IBM SPSS Version 27.0.

## Results

### Characteristics of drug shops and staff qualifications

Twenty shops were included in the survey, 10 from the rural area and 10 from the urban area. Fifteen shops sold human medicine only, 3 sold animal medicine only and 2 sold medicines for both humans and animals, both of which were in the rural area (Supplementary Table 1). In the urban area we were unable to identify any shops that sold human and animal medicines and recruited one shop selling animal medicine. All the dispensers that participated were male except one rural shop that had a female dispenser/owner.

Over-the-counter sales of antibiotics without a prescription were observed in 90% (18 out of 20) of the shops. Four shops met the requirement of staff having a Pharmacy Grade C qualification, all of which were in the urban site. Ten shop staff had undertaken rural medical practitioner (RMP) training which is not a recognised qualification for dispensing prescription medicines. Three shop staff had other qualifications (livestock artificial insemination technician, local medical assistant and family planning training, paramedic training) and three shop staff had no qualifications (two urban, one rural). Supplementary Table 1 presents the qualifications of drug shop staff in each shop type.

Five urban shops and one rural shop had a chamber (room) where a registered private doctor (MBBS) attended on a part-time basis (Supplementary Table 1). These doctors’ chambers were independent businesses that charged a consultation fee, with a separate shop sign and different opening times to the drug shop but the co-location with a drug shop was of mutual benefit. The study observation periods took place at times when the doctors’ chambers were closed.

Shops ranged in size, construction materials and storage facilities. All shops had an electricity supply. All of the urban shops had refrigerators, whereas six of the ten rural shops did not have any refrigerated storage. The most informal shops were small market stalls or sheds with corrugated iron roofing and a shop front open to the external environment. The more formal shops had glass doors and a shop front with concrete or ceramic floors situated in single story or multi-story buildings.

### Characteristics of drug shop customers

A total of 582 customers were observed; 31.6% of customers were women. In urban drug shops, women comprised almost half of all customers (47.1%) whereas in the rural shops only 17.2% of customers were women (p < 0.001) (Table 1). When broken down by shop type, the proportion of women customers was highest in shops selling human medicine (37.4%) compared to shops selling human and animal medicine (15.8%), and shops selling animal medicine only (14.0%) (Table 2).

**Table 1:** Customer prescription use and antibiotic purchasing by gender and location (urban or rural) from retail drug shop observations in Bangladesh

		Total n (%)	Urban n (%)	Rural n (%)	*p* *
Customers purchasing antibiotics (n = 574)		96 (16.7)	60 (21.6)	36 (12.2)	0.003
Customers purchasing antibiotics with prescription (n = 96)		25 (26.0)	21 (35.0)	4 (11.1)	0.01
Proportion of women and men costumers (n = 582)	Women	184 (31.6)	132 (47.1)	52 (17.2)	< 0.001
	Men	398 (68.4)	148 (52.9)	250 (82.8)	
Observations with complete purchase data (n = 574)	Women	183 (31.9)	132 (47.5)	51 (17.2)	< 0.001
	Men	391 (68.1)	146 (52.5)	245 (82.8)	
Customers with prescription by gender (all observations) (n = 78)	Women	37 (47.4)	34 (52.3)	3 (23.1)	0.002
	Men	41 (53.6)	31 (47.7)	10 (76.9)	
Customers without prescription by gender (all observations) (n = 496)	Women	146 (29.4)	98 (46.0)	48 (17.0)	
	Men	350 (70.6)	115 (54.0)	235 (83.0)	
Customers purchasing antibiotics with prescription by gender (n = 25)	Women	14 (56.0)	12 (57.1)	2 (50.0)	0.03
	Men	11 (44.0)	9 (42.9)	2 (50.0)	
Customers purchasing antibiotics without prescription by gender (n = 71)	Women	17 (23.9)	12 (30.8)	5 (15.6)	
	Men	54 (76.1)	27 (69.2)	27 (84.4)	

*using chi-square analysis

**Table 2:** Customer characteristics according to type of retail drug shop (selling human medicine, animal medicine or both human and animal medicine) from observations in Bangladesh

		Shop type			
		Human medicine n (%)	Human and animal medicine n (%)	Animal medicine n (%)	Total n (%)
All observations	Women	161 (37.4)	16 (15.8)	7 (14.0)	184 (31.6)
	Men	270 (62.6)	85 (84.2)	43 (86.0)	398 (68.4)
Customers with prescription (all observations)	No	357 (83.4)	88 (91.7)	48 (96.0)	493 (85.9)
	Yes	71 (16.6)	8 (8.3)	2 (4.0)	81 (14.1)
Customers purchasing antibiotics	No	364 (85.0)	85 (88.6)	29 (58.0)	478 (83.3)
	Yes	64 (15.0)	11 (11.4)	21 (42.0)	96 (16.7)
Customers purchasing antibiotics with a prescription	No	42 (63.6)	9 (81.8)	20 (95.2)	71 (74.0)
	Yes	22 (34.3)	2 (18.8)	1 (4.8)	25 (26.0)
Medicine sought for (all observations)	Patient (human)	239 (55.4)	49 (48.5)	3 (6.0)	291 (50.0)
	Family member	169 (32.9)	29 (28.7)	2 (4.0)	200 (34.4)
	Animal	3 (0.7)	10 (9.9)	45 (90.0)	58 (10.0)
	Practitioner or drug shop owner	12 (2.8)	8 (7.9)	0	20 (3.4)
	Not stated/missing	11 (2.6)	5 (5.0)	0	16 (2.7)
Total observations		431	101	50	582

Customers bought medicines for themselves, the patient (50.0% of all customers), a family member (34.4%), an animal or livestock (10.0%), or as a practitioner or drug shop owner (3.4%). In a small number of cases this information was not ascertained (2.7%) (Table 2). Customers seeking medicines for animals included cows, goats, hens, ducks and pigeons.

**Fig. 1:**
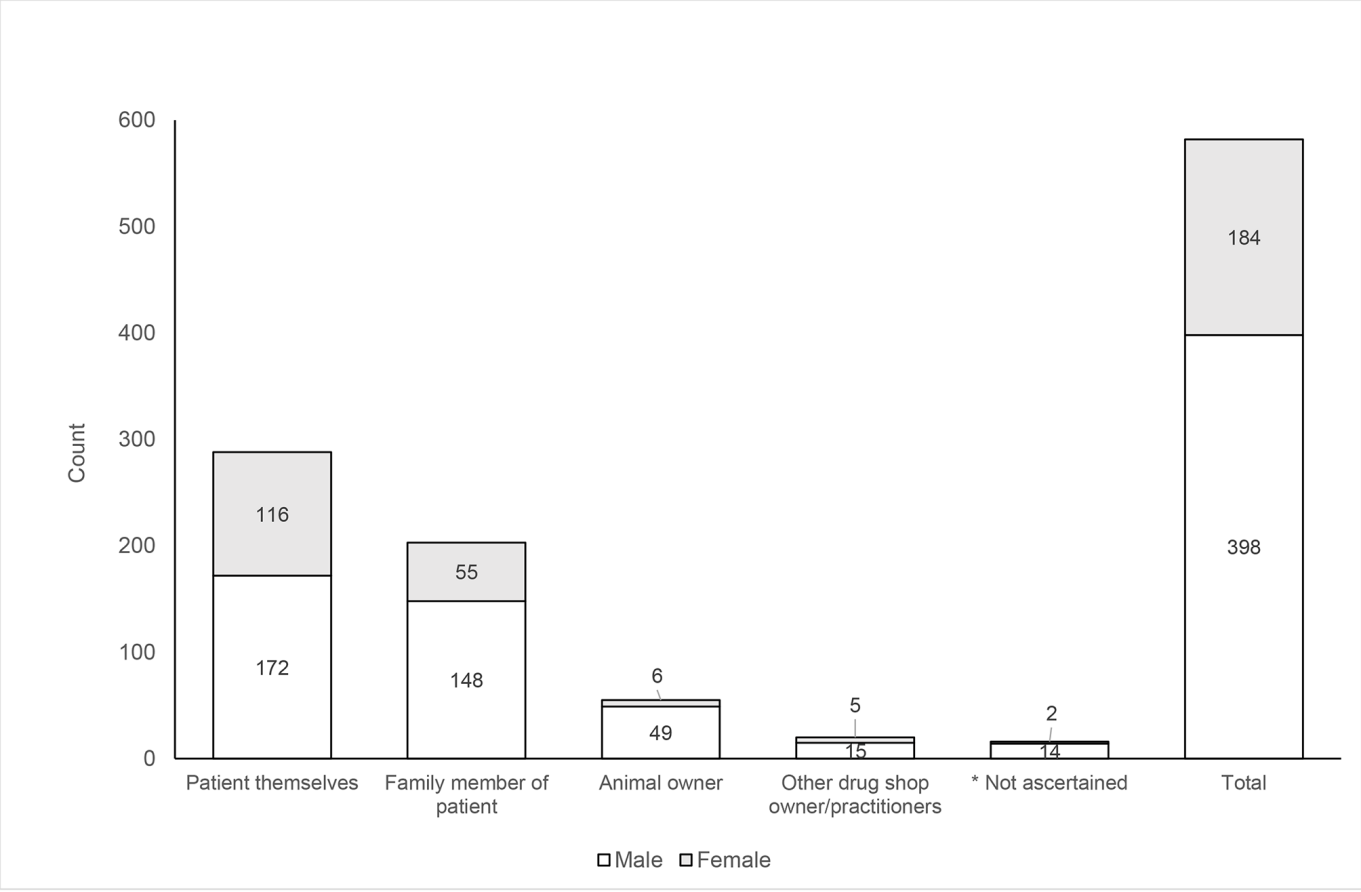
Categories of customer attending drug shops and the number of men and women within categories (n = 582 observations) *Not ascertained: denotes cases where information was not obtained

Fig. 1 shows the number of customers buying medicines for themselves (patient), another family member, for an animal or for other purposes with the breakdown of men and women in each category. Women were represented in all customer categories, but relatively few customers seeking animal medicine were women (10.9%). Further information on the types of medicine purchased in the urban and rural shops is summarised in Supplementary Table 2.

### Use of prescriptions and antibiotic purchases by gender and locality

Antibiotics were purchased in 16.7% of observations, with more antibiotic purchases in urban than in rural shops (21.6% versus 12.2% of observations, chi-square = 8.07, p < 0.003) (Table 1). Use of prescriptions for antibiotics was also more common in the urban compared to rural shops; 35.0% versus 11.1% used prescription for antibiotics respectively, p = 0.01. The proportion of customers purchasing antibiotics in different shop types, irrespective of prescription use, was highest in animal medicine shops (42.0%), followed by human medicine shops (15.0%) and human and animal medicine shops (11.4%) (Table 2). For all customer observations, women were more likely than men to have a prescription (unadjusted OR = 2.16, 95% CI 1.33, 3.51, p < 0.002), however, after adjusting for location (urban or rural), the gender difference was not statistically significant (adjusted OR = 1.34, 95% CI 0.81, 2.22, p = 0.26) (Table 3).

**Table 3:** Unadjusted and adjusted odds ratios of prescription use by gender and location (urban vs. rural) in retail drug shops in Bangladesh

			Prescription use			
			Unadjusted OR (95% CI)	*p*	Adjusted OR (95% CI)	*p*
All customers (n = 582)	Gender	Men (ref)	1.00		1.00	
		Women	2.16 (1.33, 3.51)	0.002	1.34 (0.81, 2.22)	0.26
	Location	Rural (ref)	1.00		1.00	
		Urban	5.91 (3.28, 10.62)	< 0.001	5.41 (2.95, 9.93)	< 0.001
Customers who purchased antibiotics (n = 96)	Gender	Men (ref)	1.00		1.00	
		Women	4.04 (1.55, 10.55)	0.004	3.38 (1.26, 9.09)	0.016
	Location	Rural (ref)	1.00		1.00	
		Urban	4.31 (1.34, 13.84)	0.014	3.53 (1.06, 11.71)	0.039

Among observations resulting in an antibiotic purchase, prescription use was more likely in women than men (unadjusted OR = 4.04, 95% CI 1.55, 10.55, p < 0.004) and in urban shops compared to rural shops (unadjusted OR = 4.31, 95% CI 1.34, 13.84, p < 0.014) (Table 3). The effect of gender remained significant after controlling for location (adjusted OR = 3.38, 95% CI 1.26, 9.09, p = 0.016) but the confidence intervals were wide.

**Fig. 2:**
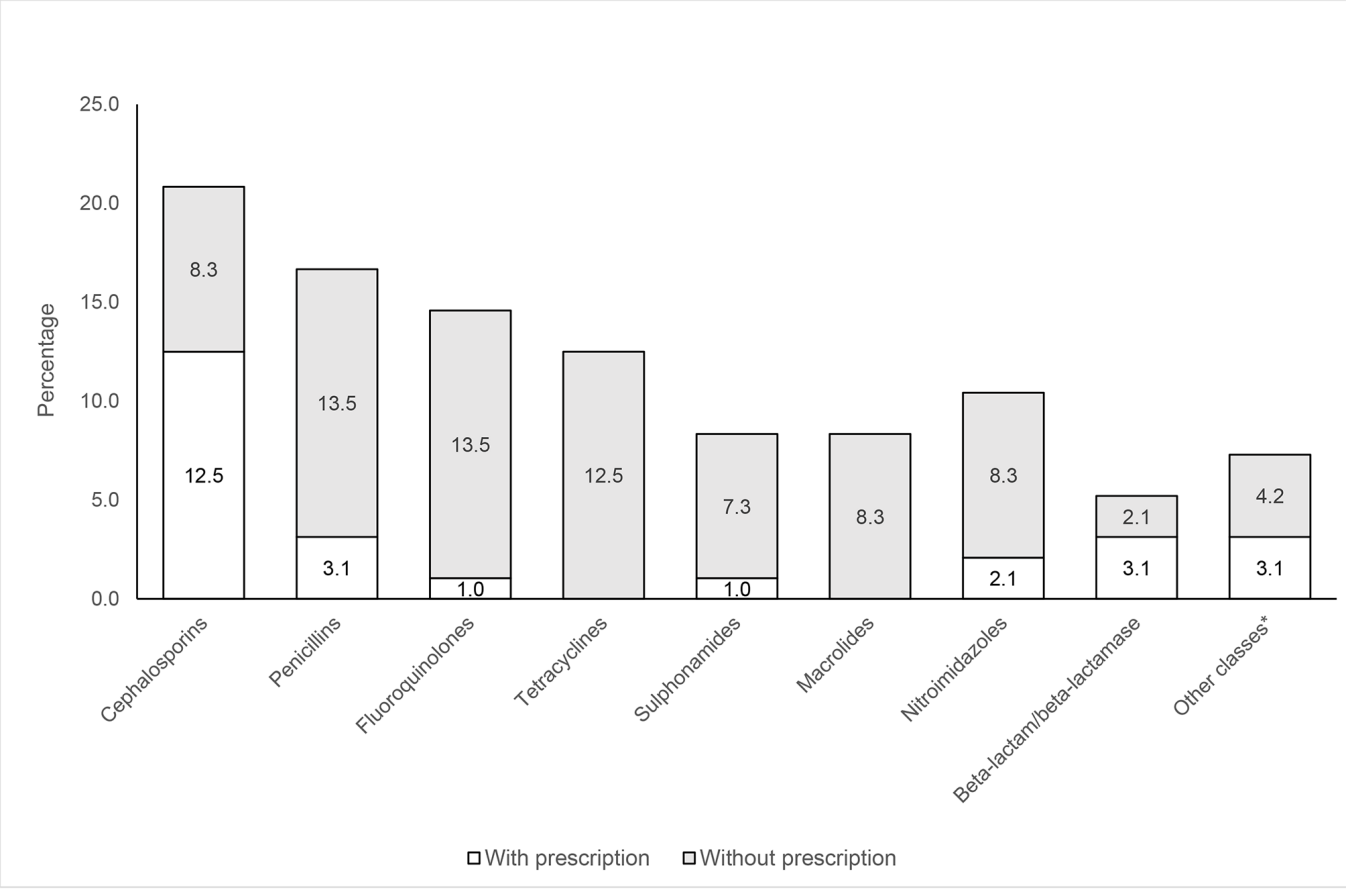
Classes of antibiotics purchased, with or without a prescription, as a percentage of total observed antibiotic purchases (n = 96) *Other classes included aminoglycosides, quinolone, mupirocin

Fig. 2 shows the classes of antibiotics for the observed antibiotics sold, and the percentage sold with or without a prescription. Cephalosporins were the most commonly dispensed antibiotic (20.8% of all antibiotics sold) followed by penicillin (16.6%) and fluoroquinolones (14.5%). Non-prescription purchases were made for all classes of antibiotics including third generation cephalosporins (ceftriaxone, cefixime, cefuroxime); penicillin (amoxycillin; flucloxacillin); fluoroquinolones (ciprofloxacin), tetracyclines (gentamycin, doxycycline, oxytetracycline); sulphonamides; macrolides (azithromycin); nitroimidazoles (metronidazole). The category of ‘other’ antibiotics included aminoglycosides, quinolone and mupirocin (topical antibiotic). Specific antibiotic classes sold with or without prescription were not broken down further by location or gender due to the overall sample size.

## Discussion

This study aimed to examine gender dimensions of drug shop attendance, antibiotic purchasing, and use of prescriptions in rural and urban areas, taking a One Health perspective by including shops for human and animal medicine. Almost all shops (90%) in the study sold antibiotics without a prescription, and only a quarter (26%) of all customers used a prescription to buy antibiotics, contrary to government regulations [20]. Although the study was limited to two study areas, findings are likely to be similar in other areas of Bangladesh. A recent study reported 100% of 189 registered retail drug shops in north and south Dhaka supplied antibiotics without prescription on request [28].

In this study, women constituted nearly one third of all drug shop customers (31.6%) but this varied by urban-rural locality. In urban drug shops, men and women attended in almost equal proportions, but in rural shops women were under-represented comprising only 17.2% of customers. These gender dimensions of drug shop use may relate to employment, income or other factors influencing female status in urban and rural areas. The urban area in this study had a high number of garment industry employers which may have contributed to women’s autonomy and health care seeking practices. Urban centres in Bangladesh, particularly those with garment industry employers, have increasing rates of female employment [29] which is, in turn, associated with increased decision-making powers and greater financial independence. Labour force participation rates for females aged over 15 years increased from 29.8% to 2010 to 36.4% in 2020 in Bangladesh, higher than neighbouring countries [30]. The ability of women to access drug shops and thereby gain direct access to health care is an important consideration of future initiatives on antibiotic stewardship.

We also observed gender differences in prescription use for antibiotics. Women were more likely to buy antibiotics with a prescription than men. However, this is in part due to the high proportion of antibiotics bought by men for animals without prescription in rural shops. Relatively few studies of over-the-counter sales of antibiotics in south and south-east Asia have provided a breakdown of customers by gender or have compared practices in urban and rural areas. Biswas et al. [31] surveyed pharmacies in three cities in Khulna division, Bangladesh where 39% of customers were women, a slightly lower proportion than we observed in urban shops. In Kerala, India, the use of prescriptions for antibiotics purchased from pharmacies increased with higher income strata and more skilled occupations, but there were no reported differences in prescription use by gender [32].

The proportion of all transactions resulting in an antibiotic purchase (16.7% overall, 21.6% urban, 12.2% rural) was lower in this study compared to customer-dispenser interactions observed in 30 pharmacies in Vietnam where 30% of urban and 24% of rural transactions included antibiotics [7]. The rates of antibiotic purchase without prescription, however, were higher in our study (74.5.%) than a survey in southern Bangladesh which reported 45.7% antibiotic purchases without prescription [18], the latter study was conducted in the rural Matlab area of demographic and health surveillance which has had a long term influence on healthcare provision in the community. We found non-prescription sale of antibiotics was higher in rural than urban shops, similar to reports elsewhere [7]. The overall proportion of customers buying antibiotics (with and without prescription), however, was higher in urban compared to rural shops, which could reflect greater financial capacity for private healthcare.

In our study, antibiotics sold from drug shops without prescription included those which are on the list of critically important antimicrobials for human medicine [33] namely ceftriaxone, cefixime, ciprofloxacin and azithromycin. The widespread and inappropriate use of broad-spectrum and critical antibiotics for human health is serious concern for antibiotic stewardship [34, 35].

Strengths of the study include the new insights gained by examining gender dimensions of antibiotic purchasing across rural and urban settings, thereby contributing to the gender equality and social inclusion (GESI) agenda. An innovation of this study was the inclusion of drug shops selling medicines for humans, animals or both. Limitations of the study include the scarcity of animal medicine shops in the urban area, hence only one was recruited to the study which may not be generalisable. Shops selling both human and animal medicine were only present in the rural area and reflect the more informal and unregulated aspects of drug shops in rural compared to urban areas. Further, we did not have an inventory of shops from which to select at random, but rather selected shops through snowball sampling, giving rise to the possibility of selection bias. Finally, the presence of a researcher during the observations may have affected dispensing practices or customer behaviours, due to the Hawthorne effect [36]. We endeavoured to minimise this effect by making the observations as unobtrusive as possible. The effect of an observer would likely lead to more appropriate practices (e.g. a lower rate of non-prescription use or fewer non-prescription antibiotics sales) and hence an underestimate of the actual rates of over-the-counter sales of antibiotics. Nevertheless, over-the-counter sales without prescription were very common. Future research with a larger sample of animal medicine shops would be a valuable addition, as well as following animal drug shop staff who visit clients on site or by telephone.

This study can help to identify priority audiences and target behaviours for improving antibiotic stewardship. A high proportion of rural drug shop owners did not have the required pharmacy qualifications, and several had no qualifications. In other studies, rural drug shops had less knowledge of antibiotic generation and appropriate prescribing, and reported less contact with medical representatives who provide information on antibiotics, compared to urban shop staff [25]. The common practice of purchasing antibiotics by proxy for a patient is another behaviour to target to prevent inappropriate antibiotic use. Rural customers buying antibiotics for animals, most of whom were men, were the least likely to use a prescription and are another important target group.

The challenge for universal health coverage and antibiotic stewardship is balancing the affordability and accessibility of drug shops against the difficulties of regulation, qualifications and risks of inappropriate antibiotic dispensing. The Bangladesh National Drug Policy aims to ensure good pharmacy practices at the point of sale of antibiotics [20]. These measures could improve antibiotic stewardship, but the lack of access to qualified healthcare professionals for those who need antibiotics is still likely to remain for a large proportion of the population. Future studies should incorporate gender dimensions of antibiotic purchase and consumption across the One Health spectrum to inform interventions and incorporate these dimensions into National Action Plans.

## Electronic supplementary material

Below is the link to the electronic supplementary material.

Supplementary Material 1

## Data Availability

The datasets used and/or analysed during the current study are available from the corresponding author on reasonable request.
