# Supplementary material for: Gender and urban-rural influences on antibiotic purchasing and prescription use in retail drug shops: a one health study
Source: BMC Public Health. 2023 Feb 2;23:229. doi: 10.1186/s12889-023-15155-3 (PMC9896778; doi:10.1186/s12889-023-15155-3)
Supplement: Supplementary file 1 — Supplementary Material 1 [file 12889_2023_15155_MOESM1_ESM.docx]

**Gender and urban-rural influences on antibiotic purchasing and prescription use in retail drug shops: a One Health study**

Emily K. Rousham, Papreen Nahar, Mohammad Rofi Uddin, Mohammad Aminul Islam, Fosiul Alam Nizame, Nirnita Khisa, S. M. Salim Akter, Mohammad Saeed Munim, Mahbubur Rahman, Leanne Unicomb

**Supplementary file**

**Supplementary table 1** Retail drug shops included in the study by shop type (human, animal or human and animal medicine), locality and qualifications of participating drug shop staff

| **Shop type** | **Location** | **Training of drug staff participant†** | **Minimum qualification requirement met** | **Other characteristics** |
| --- | --- | --- | --- | --- |
| Human medicine only | Urban | Pharmacy Grade C‡  Rural medical practitioner training | Yes | MBBS doctor’s chamber attached |
|  | Urban | Pharmacy Grade C  Rural medical practitioner training | Yes | MBBS doctor’s chamber attached |
|  | Urban | Pharmacy Grade C  Short pharmaceutical company training (half-day) | Yes | MBBS doctor’s chamber attached |
|  | Urban | Pharmacy Grade C | Yes | NA |
|  | Urban | Rural medical practitioner training | No | NA |
|  | Urban | Rural medical practitioner training | No | NA |
|  | Urban | Rural medical practitioner training | No | NA |
|  | Urban | Rural medical practitioner training | No | MBBS doctor’s chamber attached |
|  | Urban | No qualifications | No | Two MBBS doctors’ chambers attached |
|  | Rural | Rural medical practitioner training | No | NA |
|  | Rural | Rural medical practitioner training | No | MBBS doctor’s chamber attached |
|  | Rural | Rural medical practitioner training | No | Rural medical practitioner chamber attached |
|  | Rural | Rural medical practitioner training | No | NA |
|  | Rural | Rural medical practitioner training | No | Registered Model Medicine Shop, Government Model Pharmacy Initiative |
|  | Rural | Paramedic (2 year training) | No | NA |
| Human and animal medicine | Rural | No qualifications | No | NA |
|  | Rural | Local Medical Assistant and Family Planning (LMAF) (1 year training) | No | NA |
| Animal medicine only | Urban | No qualifications | No | NA |
|  | Rural | Artificial insemination technician (3 month training) | No | NA |
|  | Rural | Rural medical practitioner training | No | NA |

†Some staff had more than one qualification or training experience

‡ Pharmacy Grade C = 12 week training certificate

**Supplementary table 2:** Customer purchases in urban and rural drug shops in Bangladesh from observation data

|  | Urban  n (%) | Rural  n (%) | Total  n (%) |
| --- | --- | --- | --- |
| Antibiotics | 60 (21.4) | 36 (11.9)* | 96 (16.5) |
| Other medicine | 149 (53.2) | 156  (51.7) | 305 (52.4) |
| Painkillers, antihistamines, vitamin or mineral supplements, oral rehydration solution (ORS) | 36 (12.8) | 46 (15.2) | 82 (14.1) |
| No purchase | 23 (8.2) | 26 (8.6) | 49 (8.4) |
| Medicine not available | 5 (1.8) | 17 (5.6) | 22 (3.8) |
| Contraception/pregnancy test | 4 (1.4) | 7 (2.3) | 11 (1.9) |
| Not observed (missing) | 2 (0.7) | 6 (2.0) | 8 (1.4) |
| Antifungal | 1 (0.4) | 6 (2.0) | 7 (1.2) |
| Returned medicine | 0 (0) | 1 (0.3) | 1 (0.2) |
| Unani medicine | 0 (0) | 1 (0.3) | 1 (0.2) |
| Total | 280 (100) | 302 (100) | 582 (100) |

* Chi-square 7.74, *p* = 0.005
